# Avian Influenza A Virus Infects Swine Airway Epithelial Cells without Prior Adaptation

**DOI:** 10.3390/v12060589

**Published:** 2020-05-28

**Authors:** Dai-Lun Shin, Wei Yang, Ju-Yi Peng, Bevan Sawatsky, Veronika von Messling, Georg Herrler, Nai-Huei Wu

**Affiliations:** 1 Institute of Virology, University of Veterinary Medicine Hannover, 30559 Hannover, Germany; yangwei0727@hotmail.com (W.Y.); Ju-Yi.Peng@tiho-hannover.de (J.-Y.P.); Georg.Herrler@tiho-hannover.de (G.H.); 2 College of Veterinary Medicine, Northeast Agricultural University, Harbin 150030, China; 3 Division of Veterinary Medicine, Paul-Ehrlich-Institut, Federal Institute for Vaccines and Biomedicines, 63225 Langen, Germany; bevan.sawatsky@pei.de (B.S.); veronika.vonmessling@pei.de (V.v.M.); 4 German Center for Infection Research, Thematic Translational Unit Emerging Infections, 63225 Langen, Germany; 5 Research Center for Emerging Infections and Zoonoses, University of Veterinary Medicine Hannover, 30559 Hannover, Germany

**Keywords:** avian influenza virus, mixing vessel theory, air-liquid interface culture, innate immune response

## Abstract

Pigs play an important role in the interspecies transmission of influenza A viruses (IAV). The porcine airway epithelium contains binding sites for both swine/human IAV (α2,6-linked sialic acids) and avian IAV (α2,3-linked sialic acids) and therefore is suited for adaptation of viruses from other species as suggested by the “mixing vessel theory”. Here, we applied well-differentiated swine airway epithelial cells to find out whether efficient infection by avian IAV requires prior adaption. Furthermore, we analyzed the influence of the sialic acid-binding activity and the virus-induced detrimental effects. Surprisingly, an avian IAV H1N1 strain circulating in European poultry and waterfowl shows increased and prolonged viral replication without inducing a strong innate immune response. This virus could infect the lower respiratory tract in our precision cut-lung slice model. Pretreating the cells with poly (I:C) and/or JAK/STAT pathway inhibitors revealed that the interferon-stimulated innate immune response influences the replication of avian IAV in swine airway epitheliums but not that of swine IAV. Further studies indicated that in the infection by IAVs, the binding affinity of sialic acid is not the sole factor affecting the virus infectivity for swine or human airway epithelial cells, whereas it may be crucial in well-differentiated ferret tracheal epithelial cells. Taken together, our results suggest that the role of pigs being the vessel of interspecies transmission should be reconsidered, and the potential of avian H1N1 viruses to infect mammals needs to be characterized in more detail.

## 1. Introduction

Influenza continues to be a serious health threat for humans and animals [1,2]. The swine-origin influenza A (H1N1) virus caused a pandemic in 2009, while the outbreak of highly pathogenic avian influenza (H5N1) viruses and the emerging H7N9 and H10N8 low pathogenic avian influenza viruses continue to cause human death until now [3,4,5]. Moreover, evidence of globally widespread H9N2 viruses acquired key mammalian adaptation markers in the past decade represents a genuine threat for humans [6,7]. While avian influenza A viruses (IAVs) have the ability to infect different mammalian species even marine mammals [8,9,10], there is a need to investigate the zoonotic potential of avian influenza viruses in pigs.

Analyses of the sialic acid-binding activity in the 1980s have revealed that human IAVs preferentially use α2,6-linked sialic acid as a receptor determinant for attachment to host cells when initiating an infection [11]. By contrast, avian viruses were found to preferentially recognize α2,3-linked sialic acids. When the pig trachea was reported to contain sialic acids in both linkage types [12,13], this finding was interpreted in support of the role of pigs as “mixing vessel” for the generation of reassortant viruses [14]. The relationship between avian IAVs and influenza A viruses that are circulating in swine populations (swine IAV) worldwide is rather complicated [15]. Generally, swine IAVs may contain RNA segments of both human and avian origin via adaptation and reassortment. The classical swine IAV (H1N1) that was circulating in North America originally adapted from the human IAV H1N1, which caused the pandemic in 1918. After decades of reassortment events together with human seasonal IAVs and/or avian IAVs, a specialized lineage called triple-reassortant swine IAV (TRIG) showed up [14]. In Europe, an avian IAV of the H1N1 subtype succeeded in 1979 in establishing a new virus lineage in European pig populations [16], which was designated as Eurasian avian-like swine influenza virus H1N1 (EA-swH1N1). These viruses were predominant until 2009, when a pandemic H1N1 virus emerged as a leading cause of human death and spread also in pigs [17,18]. The sequence analysis of this virus indicated that six of the eight RNA gene segments were derived from an American swine IAV, TRIG-H1N2, while the rest originated from a EA-swH1N1 virus. Gene segments from avian influenza virus contributed to the crosspieces transmission and to the enhanced pathogenicity.

The host respiratory epithelium serves as the primary target of influenza viruses. It consists of differentiated cells with specialized properties, including ciliated cells and mucus-producing cells. Together, these cells have a barrier function preventing foreign components of the inhaled air including microorganisms to invade the host via the paracellular route. Furthermore, they provide a mucociliary clearance function by entrapping potentially detrimental substances within a layer of mucus and transporting it out of the airways [19]. As no immortalized cells have these specialized properties, primary culture systems have to be used for analyzing the course of infection by IAV. The ex vivo precision-cut lung slices (PCLS) and other organ cultures, generated from lungs of cattle, swine and chickens were used to analyze infection by respiratory viruses and bacteria [20,21,22,23]. PCLS that contain the epithelial cells in their original setting have been used to study the virulence of avian IAV in the swine airways and cross-species adaptation [23,24].

An alternative culture system is the air-liquid-interface (ALI) culture. In this system, respiratory epithelial cells are isolated and grown on collagen-coated filter supports. The cells are cultured until they are confluent, followed by the removal of the apical medium which induces the differentiation process within a time period of up to four weeks [25]. ALI cultures from human, bovine, murine, ferret and porcine airway epithelial cells have been described and used to study the infection by different respiratory viruses [20,21,26,27,28,29,30]. Recently, an immortalized human airway basal cell line, BCi-NS1.1 cells, has been reported, which can also differentiate into specialized epithelial cells, including ciliated, secretory, mucus-producing and club cells and can be applied as a human ALI culture model [31]. The selective access to both the apical and basolateral domain of ALI cultures allows studying the entry and exit route of viruses. IAV use sialic acid as the major receptor determinant on epithelial cells. The localization of sialic acids on target cells of IAV can be determined via domain-specific lectin staining and by applying antibodies directed against cell markers. One advantage of using the well-differentiated ALI culture is that the system does not contain immunocytes. In this way, the innate immune response of the epithelial cells, namely, the regulation and modulation of interferon-stimulated genes (ISGs), such as *Mx1* gene, can be studied [32,33]. Additionally, these primary systems allow researchers to reduce or even fully replace animal experiments and thus contribute to the 3R principle. By using the optimized protocol, researchers could collect the cells from the slaughterhouse or from the leftover parts from other animal experiments. The immortalized human basal cell is also a powerful tool for functional studies. With these culture systems, it will be possible to investigate the virulence properties of IAV in the airway epithelium of pigs in more detail.

In this study, we utilized the methodology of well-differentiated airway epithelial cell cultures to investigate the interspecies transmissibility of avian IAVs to mammals. Our results showed that some avian IAVs could be replicated in the swine airway epithelial cultures without prior adaptation. The receptor-binding preference of viral hemagglutinin was not the sole factor for the avian virus to infect pigs, while a weak stimulation of host innate immune response could be the strategy of avian IAVs to prolonged replication in the swine cells.

## 2. Materials and Methods

### 2.1. Viruses

Original stocks of influenza viruses, including A/Sentinel duck/Lake Constance/SRa632/2008 (avH3N2) and A/wild duck/Germany/R30/2006 (avH1N1/06) were obtained from Prof. Timm Harder and Prof. Martin Beer, Friedrich-Loeffler-Institut, Insel Riems, Germany. Swine influenza virus, A/sw/Bad Griesbach/IDT5604/2006 (swH1N1/06), was obtained from Dr. Ralf Dürrwald, Robert Koch-Institut, Berlin, Germany. A/duck/Bavaria/1/1977 (avH1N1/77) and the recombinant human R1 and R2 viruses, which contain the glycoproteins (HA and NA) of human A/Hong Kong/1/68 (H3N2) and a backbone of A/WSN/1933 (H1N1), were obtained from Dr. Mikhail Matrosovich, Philipps-University Marburg, Germany. The generation of recombinant H9N2-R66 and its mutant strain R66-HA190 were described in our previous study [24], and the original and A/chicken/Emirates/R66/2002 (avH9N2) virus together with the reverse genetic system were obtained from Dr. Jürgen Stech, Friedrich-Loeffler-Institut, Insel Riems, Germany. The recombinant viruses were generated and propagated in Madin–Darby canine kidney (MDCK) cells and virus from passage two were used for the experiments. The mammalian IAV viruses were propagated in MDCK cells, while the avian influenza viruses were propagated in the chorio-allantoic cavity of 10-day-old pathogen-free embryonated chicken eggs, aliquoted and stored at −80 °C.

### 2.2. Immortalized Cells

MDCK cells were maintained in DMEM medium containing 10% FBS. An immortalized human airway epithelium basal cell line (BCi-NS1.1), provided by Dr. Crystal (Weill Cornell Medical College, New York, NY, USA), which have the ability to differentiate into specialized epithelial cells, were maintained as previously described [31]; low passages of BCi-NS1.1 between P14 to P17 were used in this study.

### 2.3. Primary Trachea and Bronchus Epithelial Cells

The isolation procedure for primary cells from pigs (trachea and bronchus) and ferret (trachea) was adapted from the protocol of primary human airway epithelial cells isolation [34]. The lungs together with tracheas from pigs were collected from the local slaughterhouse. The tracheas from ferrets were collected from animals sacrificed for other experiments. In brief, trachea or bronchus were collected and the protease XIV together with DNase I was used to detach the epithelial cells from the mucosal parts of the epithelium. The cells were collected, and the fibroblast cells were removed by adhering to a non-treated petri dish for one hour at 37 °C with 5% CO_2_. Then the airway epithelial cells were collected and seeded on a collagen I treated cell culture flask (Nunc) until they had reached confluence. The growth medium for primary swine and ferret epithelial cells was described preciously [30]. The swine cells were tested to be free of following pathogens: porcine circovirus-2, porcine reproductive and respiratory syndrome virus, porcine cytomegalovirus, influenza A virus, porcine respiratory coronavirus, *Mycoplasma hyorhinis* and *Mycoplasma hyopneumoniae*.

### 2.4. Establishment of Well-Differentiated Airway Epithelial Cell Cultures

When establishing well-differentiated ALI cultures, primary swine trachea/bronchial epithelial cells were transferred to type IV collagen coated Transwell^®^ polycarbonate membrane and grown into well-differentiated swine ALI (swALI) as previously described [30]. Primary ferret trachea epithelial cells were transferred to Matrigel^®^ coated Transwell^®^ polyester membranes with a pore size of 0.4 μM (Corning, Corning, NY, USA; the procedure was adapted from primary swALI culture method as above. The ferret-ALI medium consisting of a 1:1 mixture of DMEM/F12 (Gibco, Paisley, UK) and AECGM (Promocell, Heidelberg, Germany) with additives was described previously in the primary cell growth medium section. After the cells had reached confluence, the medium from the apical surface was removed, and the culture was maintained under air-liquid interface conditions for at least three weeks at 37 °C in 5% CO_2_ until the cells were well-differentiated. The immortalized BCi-NS1.1 human basal cells grew as ALI cultures into well-differentiated cells as described [31], and the ALI medium was a 1:1 mixture of DMEM (Gibco) and AECGM (Promocell) together with the same additives as the growth medium [34].

### 2.5. Swine Precision-Cut Lung Slice Preparation

The PCLS system has the advantage that the cells are retained in the original cell setting and that non-epithelial cells, such as immune cells, are also present. In brief, lungs from pigs were collected from a local slaughterhouse and transferred on ice directly to the laboratory. After a gross pathological examination, the lobes of the lung were filled with 1.5% low-melting agarose. After solidification, round-shaped slices, 8 mm in diameter and 250 μm thick, were generated by the Krumdieck tissue slicer. The tissue samples prepared in this way contained a terminal bronchus in the center of the slice. Prepared lung slices were cultured at 37 °C with 5% CO_2_ in RPMI1640 medium (Genaxxon, Ulm, Germany) containing antibiotics as previous described [35].

### 2.6. Cilia Vitality Assay after Influenza Virus Infection

Prior and after influenza A virus infection, PCLS were examined under the light field microscope. Examined bronchioles were divided into ten sections, and the cilia beating activity was determined for each part. The beating activity was scored from 1 to 10, and the sum for each slice was recorded. Only slices with values of more than 95% of cilia remain beating activity were used to perform infection experiments. The slices were washed twice with PBS, and 5000 focus forming units (FFU) of virus were diluted in 250 μL DMEM containing *N*-acetyl-trypsin (NAT, Sigma, Saint Louis, MO, USA) and 1% BSA. After one hour of inoculation, the infection medium was replaced by 1 mL RPMI1640 (Genaxxon) as previously described [35]. Cell supernatant (100 μL) was collected daily, and the virus replication kinetics were determined by focus-forming assay.

### 2.7. Influenza Virus Infection of ALI Cultures

Prior to influenza A virus infection of the well-differentiated ALI cultures, the apical surfaces were washed five times with sterile PBS. DMEM (100 μL) containing 125,000 FFU of virus (MOI 0.25) was applied on the apical side for 2 h of incubation time at 37 °C. At the time of measurement, apically released viral particles were collected by overlaying the cells with medium for 30 min for virus harvesting [30]. The virus replication kinetics were determined by performing a focus-forming assay.

### 2.8. Lectin Staining

Lectin staining of cells was performed by applying the sialic acid-specific agglutinins from biotinylated Maackia amurensis (MAA, α2,3-linked sialic acid, Vector laboratories) and FITC conjugated Sambucus nigra (SNA, α2,6-linked sialic acid, Vector laboratories) to the apical surface of ALI cultures. The binding of biotinylated lectins was visualized by fluorescence microscopy using streptavidin-Cy3 (Sigma).

### 2.9. Immunofluorescence Analysis of Well-Differentiated ALI Cultures

ALI cultures on the transwell supports were fixed with 3% paraformaldehyde and permeabilized by 0.5% Triton X-100. The cells were blocked with 1% BSA to avoid non-specific binding and incubated with anti-influenza A virus nucleoprotein (NP) antibody (Bio-Rad, Oxford, UK) followed by Alexa Fluor^®^ 488 conjugated secondary antibody staining. Ciliated cells were stained by Cy3-labeled antibody against β-tubulin (Sigma). A counterstain for the nuclei with DAPI (4′,6-diamidino-2-phenylindole) was performed. ProLong^®^ Gold Mountant (Life Technologies, Eugene, OR, USA) was used for embedding. Immunofluorescent imaging and examination were performed by using a Leica TCS SP5 AOBS confocal laser scanning microscope. LAS AF Lite software version 2.6.3 (Leica, Mannheim, Germany) and ImageJ/Fuji software version 1.50e (National Institutes of Health) were used for processing and analyzing. Images of three infected filters (four random fields of each filter) were used to determine the coverage of ciliated cells.

### 2.10. Determination of Virus Infectivity

For determining virus titers, 100 μL DMEM were inoculated on the apical surface of ALI culture for 30 min. Collected inoculum were aliquots and were stored at −80 °C. Virus titers were determined on MDCK cells as FFU as described previously [33]. Briefly, MDCK cells were seeded in 96-well plates, and serial 10-fold dilutions of ALI culture inocula in DMEM containing 5 µg/mL NAT were added. After incubation for 24 h at 37 °C, cells were washed, fixed with 4% formalin and permeabilized with quencher buffer (0.5% Triton X-100 with 20 mM glycine in PBS), followed by incubation with a primary anti-influenza polyclonal antibody (Virostat #1301) and a secondary HRP antibody (SeraCare KPL). Subsequently, substrate (True Blue, SeraCare KPL) was used for immunological staining. Foci were counted and calculated as FFU per transwell support. The detection limit of the assay was 200 infectious particles. Thus, for samples where no foci were detected, data points were set to 100 FFU.

### 2.11. RT-PCR for ISG Transcript Analysis

Melting curve analysis with real-time PCR was performed to determine the expression level of messenger RNA in treated or infected ALI cultures. Cells were infected apically with MOI 0.25 (1.25 × 10^5^ FFU) in 100 µl DMEM. On 24 h p.i., the transwell supports were prepared, washed in PBS and stored in 300 µl RNA Later (Invitrogen, Paisley, UK). Subsequently, total RNA was prepared using RNeasy Minikit^®^ according to the manufacturer instructions (Qiagen). 1 µg of total RNA was reverse transcribed into cDNA anchored with poly-dT primer by using SuperScript III reverse transcriptase (Invitrogen) according to the manufacturer instructions. Subsequently, the real-time PCR reaction was performed in a Mx3005p Real-Time PCR system (Stratagene, Agilent) using QuantiTect SYBR Green PCR Kit (Qiagen) according to the manufacturer’s instructions. Primers and references for detecting swine Mx1, ISG15, IFNβ, and β-actin transcripts were listed in Table 1. β-actin were used as housekeeping gene control. Specificity of the PCR amplification was assessed by the melting-curve at the end of the reaction. Relative expression levels of selected genes were calculated by the 2^−ΔΔCT^ method and calculated as fold change induction compared to mock infected controls [36].

### 2.12. JAK/STAT Signal Pathway Inhibition Analysis

One day prior to infection, swine ALI cultures were treated with 1 µg of poly (I:C) (Polyinosinic:polycytidylic acid, InvivoGen) in 100 µL of DMEM medium for two hours in 37 °C by apical application. The control group received 100 µL of DMEM. For analyses of the JAK/STAT signaling pathway, swine ALI cultures received ALI medium containing 2.5 µM of Ruxolitinib (Invivogen) prior to influenza infection as previously described [37]. Swine ALI cultures with Ruxolitinib and/or poly (I:C) pre-treatment were infected by avH1N1/06 or swH1N1/06 via apical application as described above. For the JAK/STAT inhibition groups, the Ruxolitinib was continuously supplied in the ALI medium during the experiment. At 24 h post infection, the cells were collected, and total RNA was extracted as described above. 1 µg of total RNA was reverse transcribed into cDNA anchored with Uni12 primer as described above. 2 µL of cDNA product was amplified with specific primers (M52C and M253R) to amplify the Matrix gene of IAV as described previously [38]. Quantitative PCR and melting curve analyses were carried out by Quantitect SYBR Green PCR kit (Qiagen) in a Mx3005p Real-Time PCR system. PCR products exhibiting melting peaks at 85 ± 0.5 °C were counted as positive [39]. The relative quantity of viral M gene in the ALI culture was calculated by 2^−ΔΔCT^ method by normalizing to non-ruxolitinib/non-poly (I:C) treated group.

**Table 1:** Primers used for quantitative real-time PCR.

Target Gene	Primer Name	Sequence	Reference
Swine Mx1	sMX1-F1	AGCGCAGTGACACCAGCGAC	[40]
	sMX1-R1	GCCCGGTTCAGCCTGGGAAC	[40]
Swine IFNβ	sIFNb-F1	AGTTGCCTGGGACTCCTCAA	[41]
	sIFNb-R2	CTGAGAATGCCGAAGATCTG	[42]
Swine ISG15	sISG15-F1	GGTGCAAAGCTTCAGAGACC	[40]
	sISG15-R1	GTCAGCCAGACCTCATAGGC	[40]
Swine β-Actin	SusBetActin-L	GACATCCGCAAGGACCTCTA	[43]
	SusBetActin-R	ACACGGAGTACTTGCGCTCT	[43]
Influenza M gene	M52C	CTTCTAACCGAGGTCGAAACG	[38]
	M254R	AGGGCATTTTGGACAAAKCGTCTA	[38]
	Uni12	AGCAAAAGCAGG	[44]

### 2.13. Statistical Analysis

Data and statistical analysis were performed using GraphPad Prism (GraphPad Software version 5, San Diego, CA, USA). Results were presented as means ± SEM. Statistical significance between groups was determined using the one-way ANOVA with Tukey’s post-hoc test. Imaging analysis of cilia coverage rates to the well-differentiated cells was calculated by using ImageJ. The immunofluorescent images were taken by Lecia SP5. Genetic analysis was performed via the website platform of GISAID EpiFlu genebank.

## 3. Results

### 3.1. Avian Influenza Viruses Infect Swine Airway Epithelium in Different Patterns

**Figure 1:**
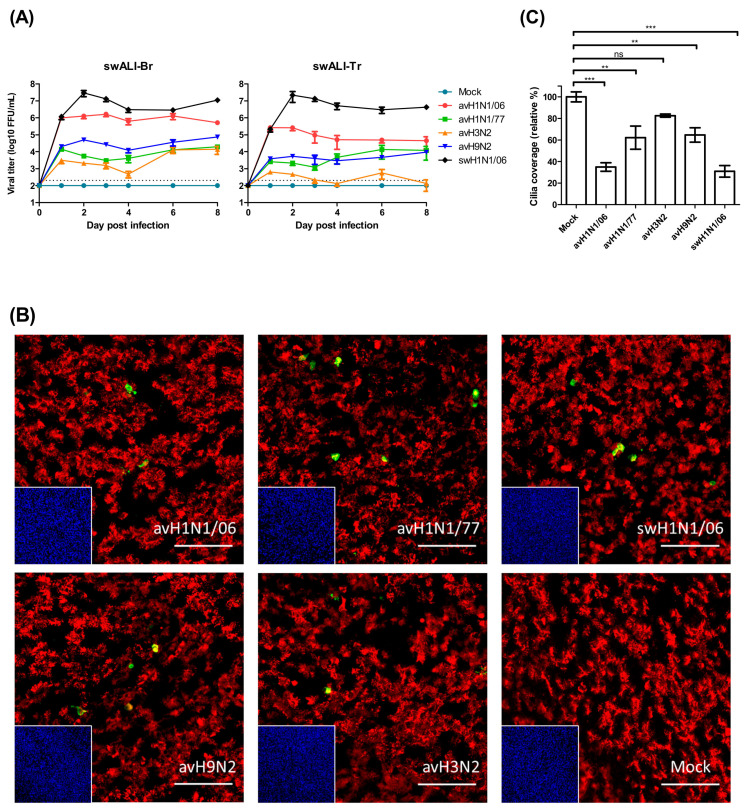
Avian influenza viruses showed different patterns of infectivity on infected swine airway epithelial cell (swALI). (**A**) Primary bronchial epithelial cells (swALI-Br) and primary trachea epithelial cells (swALI-Tr) were infected by different subtypes of avian influenza A viruses (IAVs), including avH1N1/06, avH1N1/77, avH3N2 and avH9N2. The swH1N1/06 was included in the experiment to compare the infectivity and the pathogenicity. The apical inoculum was collected for eight days, and the titer of released infectious viral particles were measured by using focus forming assy. The dashed lines indicate detection limits for the assays. (**B**) Target cells of the avian influenza viruses were located by using immunofluorescence staining. Infected swALI were fixed at 12 h post infection to show the target cells of avian IAVs. Red: ciliated cells, Green: virus NP protein, Blue: nuclei, Yellow: colocalization. Scale bars, 100 μm. (**C**) Detrimental effect of avian IAVs on swALI. The cells were fixed at 8 days post infection to determine the loss of cilia after avian IAV infection. The average coverage of ciliated cells was measured by using ImageJ/Fuji and shown in bar chart; mock infected group was normalized to 100%. The results were shown as means ± SEM. ** *p* < 0.01 or *** *p* < 0.001 in one-way ANOVA with Tukey’s post-hoc test, respectively. ns denoted as not significant (*p* > 0.05).

Primary swine airway epithelial cells were infected by avian IAVs of different subtypes or swine H1N1 virus to compare the efficiency of infection in porcine cells. SwALI cultures derived from the trachea or bronchi were grown under air-liquid interface conditions for at least 3 weeks until they were fully differentiated (ciliated cell coverage approximately 80–90%). For our infection studies, avH1N1/77, avH1N1/06, avH3N2, avH9N2 and swH1N1/06 were applied to the apical surface of the swALI cultures for a two hours inoculation period. On the day of virus harvest, 100 µL of DMEM were applied to the apical surface and incubated for 30 min to collect the released virus particles. The virus replication kinetics were determined via focus forming assay with MDCK cells. The results show that, on the first day post infection, most of the avian IAVs display a lower virus titer on swALIs compared to swH1N1/06 virus. Surprisingly, the avH1N1/06 virus reached a high titer of released infectious virus (10^6^ FFU) on the first day after infection, which showed no significant differences when comparing to the virus titer of swH1N1/06 virus-infected group (Figure 1A). Virus release at this high level was maintained for a period of up to eight days p.i. (Figure 1A).

To determine the cell tropism of avian IAVs, the differentiated cells of the swALI cultures were subjected to immunofluorescent staining as described previously [30]. At 12 h post IAV infection, the swALI were fixed and analyzed for the presence of virus antigen (nucleoprotein NP) and β-tubulin, the major component of cilia. As shown in Figure 1B, all avian IAVs used in this study were mainly detected in ciliated cells.

To get information about the detrimental effects of the infection by avian IAVs, we determined the loss of ciliated cells by monitoring changes in β-tubulin staining at eight days p.i., and the coverage of ciliated cells was analyzed. As shown in Figure 1C, infection by any of the viruses resulted in a partial reduction of the cilia coverage. The loss of ciliated cells was most pronounced after infection by the avH1N1/06 virus. In fact, the value of 34.9 % was close to that determined for the swine virus, 30.9%.

### 3.2. avH1N1 Can Infect Swine Lower Resipartory Epithelial Cells without Prior Adaption

**Figure 2:**
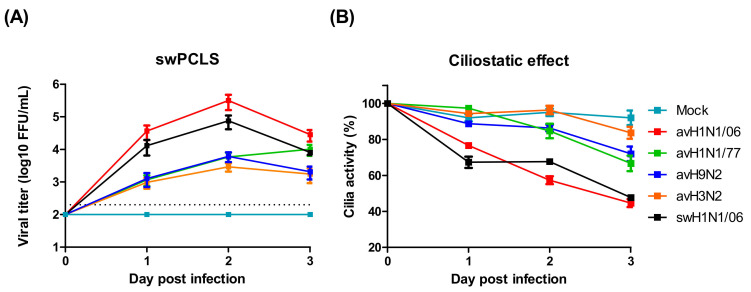
Avian influenza viruses infect swine lower respiratory tract. (**A**) Swine precision-cut lung slices (swPCLS) were infected by different avian IAV subtypes. The avH1N1/06 shows high infectivity in the swPCLS, while other avian IAVs contain low infectivity. The dashed lines indicate detection limits for the assay. (**B**) The cilia activities of the infected swPCLS. The cilia beating activity reduced after virus infection. The maximal detrimental effect was observed in avH1N1/06 infected group with approximal 60% on day 3 post infection. The results were shown as means ± SEM.

An important characteristic of virulent IAV is the ability to spread infection from the upper to the lower respiratory tract. We used swine PCLS to investigate whether the avian IAV are able to infect the epithelium of the lower respiratory tract of pigs. Slices derived from bronchial tissue were infected by either of the viruses used in this study. At daily intervals, samples were collected to determine the virus titer. At each of the three days analyzed, the highest virus titer was determined for the avH1N1/06 virus even 2.8-folds higher than that of the swine virus. The values of the other avian IAVs were more than hundred-fold lower than those of avH1N1/06 and swH1N1/06 (Figure 2A). In the PCLS system, the detrimental effect of the infection can be conveniently evaluated by monitoring the ciliary activity. In the mock infected group, ciliary activity dropped only slowly over time. Similar to the replication kinetics, the virus-induced ciliostatic effect was most pronounced in PCLS infected by avH1N1/06 where, upon microscopic inspection, more than 50% of the airway surface were lacking ciliary activity on day 3 after infection. In contrast, avH3N2 virus showed the lowest effect with a ciliary activity similar to that of uninfected slices (Figure 2B).

### 3.3. Sialic Acid Binding Preference Has a Limited Influence on IAV Infection of the Swine Airway Epithelium

**Figure 3:**
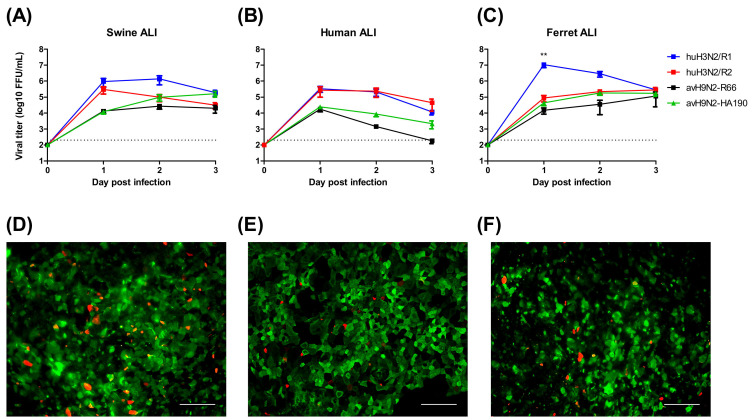
Sialic acid dependence infectivity of IAVs on well-differentiated ALI cultures. Well-differentiated ALI culture from primary swine trachea epithelial cell (**A**,**D**), human airway epithelium basal cell BCi-Ns1.1 obtained by total airway epithelium brushing (**B**,**E**) and primary ferret trachea epithelial cells (**C**,**F**) were infected by avH9N2-R66 and its mutants HA190 or recombinant human H3N2 R1 and R2 viruses (**A**–**C**). Lectin staining for detecting sialic acid expression on the apical surface of well differentiated ALI cultures showed in the lower panel (**D**–**F**). Antibodies against SNA (green) and MAA II (red) were used to recognize α2,6- and α2,3-linked sialic acids, respectively. The results were shown as means ± SEM. Scale bars, 100 μm. ** *p* < 0.01 in one-way ANOVA with Tukey’s post-hoc test.

Pigs are considered as an intermediate host for avian IAVs in the transmission to humans based on the assumption that the sialic acid distribution of the airway epithelium is favorable for both avian and human viruses. To analyze the importance of sialic acid binding activity for the transmission to new hosts, we used recombinant IAVs, which had previously been shown to differ in their binding preference for cell surface sialic acids. Avian H9N2-R66 preferentially recognizes α2,3-linked sialic acid, whereas its mutant strain HA190 (A190V mutation in the HA protein) binds most efficiently to α2,6-, α2,8- and α2,9-linked sialic acids [24]. In addition, recombinant human H3N2 virus R1, and its mutant strain R2 were used, which also differ in their binding activity, R1 having a preference for α2,6-linked sialic acid R2 binding to α2,3-linked sialic acid. These viruses were used to infect ALI cultures derived from different species, human, swine and ferret. Lectin staining revealed that the distribution of sialic acids on the well-differentiated airway cells was similar, with a predominant presence of α2,6-linked sialic acid and a low expression level of α2,3-linked sialic acid on the apical surfaces (Figure 3D–F). The result of the infection of these ALI cultures by the different viruses is shown in Figure 3A–C. In general, the two human viruses (R1, R2) replicated to higher titers than the two avian viruses (R66, HA190). The change in the receptor binding activity of the respective viruses from preferential recognition of α2,3 to α2,6-linked sialic acid increased the infection efficiency only to a minor extent. A major difference between R1 and R2 virus was found in ferret ALI; however, such a difference was not observed between the two avian viruses.

### 3.4. Different Patterns of the Innate Immune Response Alter the Infection by H1N1 Viruses

**Figure 4:**
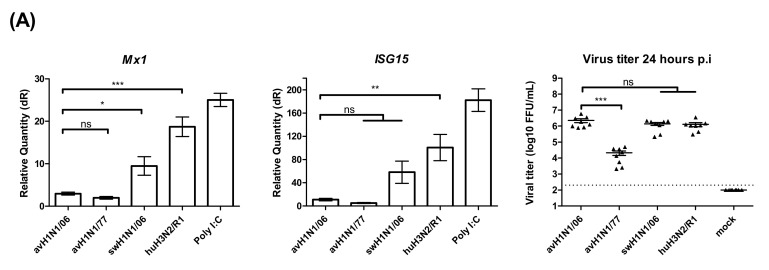

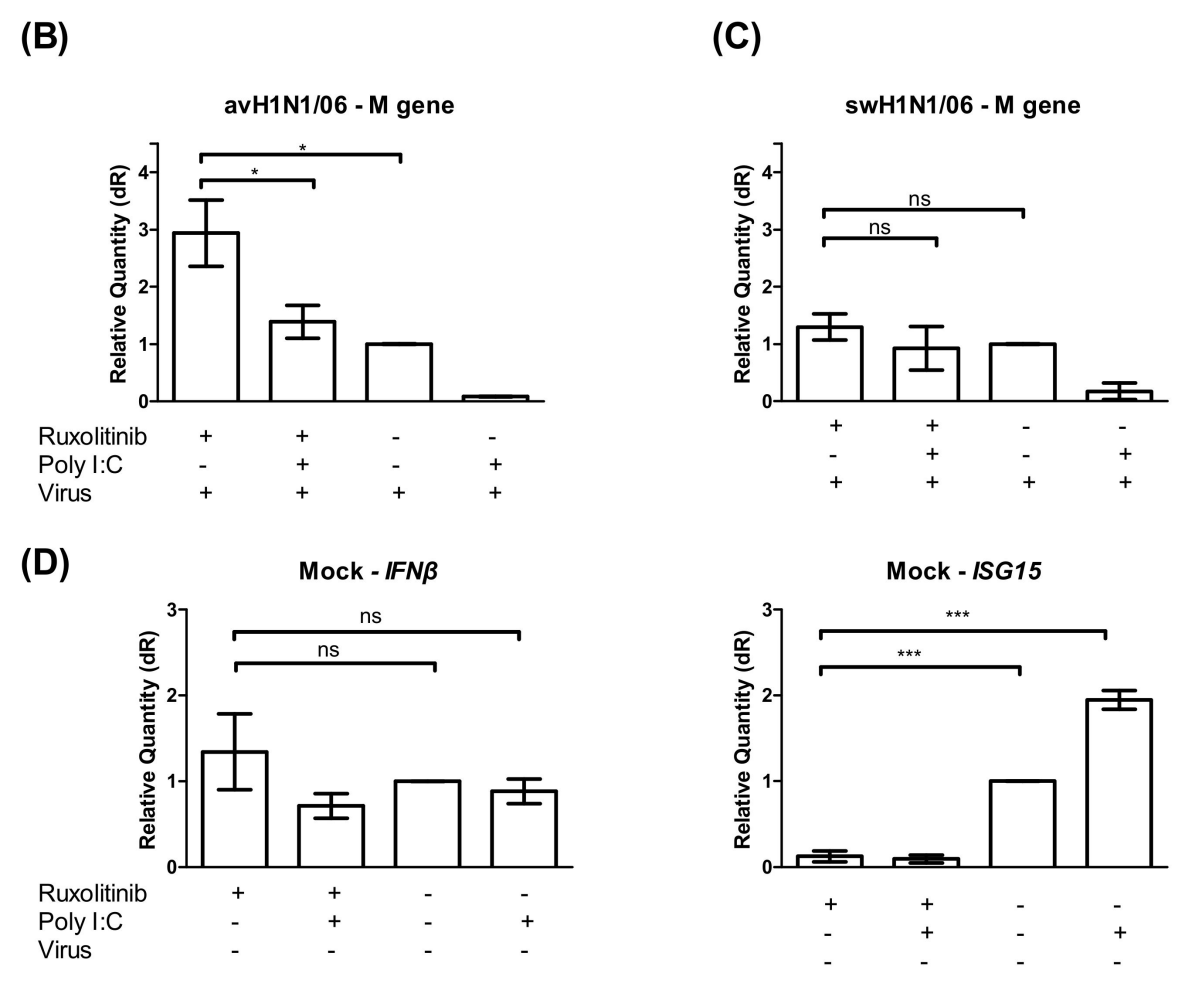
Innate immune response in swALI after IAV infection. (**A**) Relative quantity of *Mx1*, *ISG15* genes on swALI after IAV infection. swALI were infected or Poly (I:C) pretreated for 2 h from the apical surface. On 24 h post-infection, the messenger RNA from each group was used to perform quantitative real-time PCR. *Mx1* and *ISG15* genes were normalized to β-actin expression. Calculation of 2^−ΔΔCT^ method was normalized to mock infected groups. Results showed avH1N1 viruses represented as weak inducers for *ISGs*. (*n* = 8 total samples from three individual animals). On 24 h post-infection, the released viral particles from each group were determined by focus-forming assay and presented as scatter plot. (**B**,**C**) Viral M gene of influenza A virus expression pattern of swALI after avH1N1/06 (**B**) or swH1N1/06 (**C**) infection. The cell cultures were pretreated with/without JAK/STAT inhibitor and/or Poly (I:C). In ruxolitinib pretreat group, avH1N1/06 infected cells showed increased viral M gene expression. As for the swH1N1/06 infected cells, the viral M gene shows similar expression level in with or without ruxolitinib pretreatment. (**D**) This panel showed the *ISGs* expression level of mock infection cells on 24 h post infection. After pre-treating with ruxolitinib, either PBS and/or Poly (I:C) treatment induce low amount of *ISG15* expression, while the *IFNβ* remained stably expressed. The results were shown as means ± SEM. * *p* < 0.05, ** *p* < 0.01 or *** *p* < 0.001 in one-way ANOVA with Tukey’s post-hoc test, respectively. ns denoted as not significant (*p* > 0.05).

Different factors may affect the infectivity and replication kinetics of avian IAV in swine epithelial cell cultures. The innate immune response to virus infection that can be measured in air-liquid interface cultures is a straight forward target to look at. The relative expression of the interferon stimulated genes (ISGs), such as the upregulation of *Mx1* and *ISG15* genes, was used to reflect the host responses to IAV infected cells. At 24 h post virus infection, the cells were collected from filter supports, and the total RNA was extracted. Two steps quantitative real-time PCR results showed that the ISGs were upregulated at a lower degree in avH1N1/06 and avH1N1/77 infected cells. The recombinant human H3N2/R1 infected cells strongly upregulated the Mx1 expression similar to the Poly (I:C) control, while the swHN1/06 virus stimulated ISGs to an intermediate level (Figure 4A). The amount of infectious viral particles released from each tested filter supports were determined by focus-forming assay. Interestingly, avH1N1/06 stimulated a low amount of ISGs though the virus replicated efficiently (Figure 4A) and caused detrimental effect to the swine ALI cultures on day one post-infection (Figure 1C). In order to understand how the innate immune response modulated the replication of avH1N1/06, we inhibited the JAK/STAT signaling pathway together with a stimulation of the interferon expression to monitor the effect on the virus replication process. The swALI cultures were pretreated with JAK/STAT pathway inhibitor Ruxolitinib 2.5 μM prior to and during the infection to inhibit the innate immune response. Subsequently, the swALI cultures were treated with or without poly I:C 1 μg/well one day prior to infection to stimulate autocrine or paracrine signaling. Comparing the virus M gene expression at 24 h post- infection showed that the virus replication of avH1N1/06 could be inhibited by paracrine interferon stimulation, while in the Ruxolitinib pretreated group, virus replication was enhanced and the autocrine stimulation of ISGs modulated to a lower extent (Figure 4A,B), suggesting that avH1N1/06 is a weak ISG stimulator in swine airway epithelial cells. Interestingly, when the swALI were infected by swH1N1/06, the Poly (I:C) pretreatment to the cells slightly inhibited the virus replication via the paracrine interferon stimulation (without Ruxolitinib in Figure 4C), whereas the autocrine and intrinsic stimulation of ISGs showed no influence on the virus replication when the downstream of JAK/STAT signal pathway was blocked (cells with Ruxolitinib in Figure 4C). *IFNβ* and *ISG15* stimulation after mock infection had been shown in Figure 4D. A lack of efficient inhibition to the virus replication pointed out that the swH1N1/06 may escape from the innate immune modulation, which bypass the Mx1 or other ISGs antiviral function. These results indicate that different viruses use different strategies for overcoming the innate immune effects and for replicating to high titers in swine airway epithelial cells.

## 4. Discussion

A hallmark of the epidemiology of IAV is the high genetic variability that enables the virus to escape the host immune defense. Point mutations in the two surface glycoproteins, haemagglutinin (HA) and neuraminidase (NA), are responsible for minor antigenic changes observed in new strains that emerge each year and replace the preceding ones. In contrast to this antigenic drift, from time to time, there may also occur major antigenic changes. They are designated antigenic shift and are the result of reassortment of viral gene segments in cells co-infected by two influenza A viruses originating from different species. If the reassortant virus contains a hemagglutinin or even both surface glycoproteins (HA and NA) from the non-human viral partner, the human population does not have any immune protection and the virus can spread more easily than is observed with viruses within an antigenic drift period. These observations raise the question concerning the mechanisms underlying these processes, i.e., how avian influenza virus overcomes the barrier between the avian and the human species.

The hemagglutinin (HA) protein is important for influenza A viruses to attach to target cells and to initiate the infection process. Sialic acids expressed on surface components of target cells are the major attachment sites for IAV. Avian IAVs mainly bind to α2,3-linked sialic acid, which enables the infection route via the intestine and the airway of poultry [45,46]. In contrast, human IAVs preferentially bind to α2,6-linked sialic acid [12]. The porcine trachea contains sialic acids in both linkage types [47]. In recent years, the technology available for identifying sialic acids on host tissues improved and enabled a detailed analysis of the different portions of the airways with respect to sialic acid expression. These results revealed that the distribution of sialic acid linkage types in the airways of pigs is very similar to that in the human counterpart: The upper airways contain mainly α2,6-linked sialic acid, and in the lower airways, the proportion of α2,3-linked sialic acid increases [48,49]. Therefore, the sialic acid distribution in the airway does not favor pigs over humans in generating reassortant viruses with gene segments from avian and mammalian IAV. More information about the co-infection of differentiated airway epithelial cells by different influenza A viruses is required to get a better understanding of the generation of reassortant viruses. One of the questions to be answered is whether the avian IAV gain benefit from the sialic acids expressed on the airway epithelium.

To elucidate how the infection by IAV is initiated in mammals, we conducted infection experiments with differentiated airway epithelial cells and with recombinant IAVs from different origins. By analyzing the pair of avian and the pair of human viruses, each differing only in the sialic acid binding activity, we were able to understand to what extent the infection characteristics are determined by the sialic acid binding activity. Our results using the recombinant strains did not show significant differences in the infectivity nor in the virus replication rate in the infected swine or human cells. Our results show that the sialic acid-dependent binding affinity only has a minor effect on the ability of IAV to infect swine or human airway epithelial cells. Both human viruses replicated to higher titers than did the two avian viruses. For both the human and the avian viruses, the switch in the preferential binding activity from α2,3-linked sialic acid to α2,6-linked sialic acid only, resulted in a minor advantage. This result shows that the sialic acid binding preference is not the major player for avian IAV in the infection of swine and human airway epithelial cells.

A slightly different outcome was observed in the infection experiment with the ferret ALI culture. Here, only the human virus with α2,6-linked sialic acid binding activity showed efficient infection. Several IAV studies, for instance, with the highly pathogenic avian influenza virus H5N1 [50,51] or pandemic swine-origin H1N1 virus [52], had used the ferret animal models to study airborne transmission. Their results showed that the receptor-binding activity shift from α2,3- to α2,6-linked sialic acid-binding preference is crucial for the viruses to gain the ability of airborne transmission. Thus, factors critical for replication of avian viruses in ferrets are different from those determining infection in pigs.

Avian IAV of the H1N1 subtype have been suggested as the potential zoonotic agents that may be able to initiate the next influenza pandemic in humans [53,54] or infect other mammals [55]. Various zoonotic gene markers were described in the ferret model related to an enhanced ability of avian H1N1 to cross the species barrier and/or to air-bone transmission. Following alignment comparison between the viruses from published studies and the viruses we used in this study, we could not locate the exact factors that contribute to the virulence of the avH1N1/06 in our swine ALI cultures and also in the PCLS system. One explanation might be that the swine airway epithelium not only expresses both types of sialic acid receptor, but it may also provide favorable conditions for efficient replication or for proteolytic activation of the viral hemagglutinin. Cellular proteases cleave the HA0 protein into the subunits HA1 and HA2 particles and thus render the virus fusogenic, which is required for initiating the virus entry process. TMPRSS2 (transmembrane protease, serine 2) functions as the most important protease for activating H1N1 viruses and some coronaviruses including SARS-COV-2 that emerged in 2020 [34,56]. Similar to the study described elsewhere [57], the TMPRSS2 and TMPRSS4 were stably expressed in our swine ALI culture and PCLS, while TMPRSS11d (swAT) was expressed at a lower level in PCLS (data not shown). Since influenza virus subtypes other than H1N1 may require different proteases for priming virus entry [58], the different expression level of proteases may explain why some avian IAV strains showed low infectivity in our swine PCLS infection experiment.

One has to point out that the theoretical ancestor of EA-swH1N1 was the avH1N1/77, which we used in this study [15]. Unlike what we had expected, the avian H1N1 isolate from 1980s, avH1N1/77, did not replicate efficiently in the swine cells. This may be due to the fact that essential mutations for replication of avian H1N1 viruses in swine cells have not yet occurred in the avH1N1/77 strain. For example, previous genetic analysis data have shown that the components of the viral RNP complex of the EA-swH1N1 virus may have been acquired from other avian IAVs [15]. Another point to be considered is that assembling of the viral RNP with a nuclear protein mutated in the position R99K has been shown to help the EA-swH1N1 to escape the antiviral function of Mx1 [59].

The pseudostratified airway epithelium mainly relies on the innate immune system to counterattack invading viruses. The interferon-dependent response, such as the Mx1 binding to the viral RNP is an easy and straight forward reaction of the host cells [32,60]. When the cells are infected by influenza A virus, a short fragment of RNA can stimulate RIG-I and initiate expression of IFN that will be released to the basolateral side of the cells on the transwell support. After IFN has bound to IFNAR, the cascade of JAK/STAT signaling will provide a direct mechanism to translate the ISG proteins, for example, Mx1 and ISG15 [61]. Although some studies claimed that the swine respiratory epithelium induces a low amount of ISGs and inhibits the virus replication inefficiently [62], the efficient replication of avH1N1 is only associated with a relatively low ISGs stimulation. Virus amplification has been observed over a prolonged period of time. How the virus overcomes the innate immune response needs to be clarified in the future. In our study, when the swALI culture was pretreated with poly I:C, a RIG-I antagonist that stimulates a high level of interferon-dependent response, virus replication was reduced by about 100-fold. Here, we established a novel method by applying the JAK/STAT signal pathway inhibitor to the ALI culture for distinguishing whether the virus acts as a strong immune modulator or a weak stimulator for the innate immune response. We pretreated the swine ALI culture with ruxolitinib, a potent universal JAK/STAT pathway inhibitor, and successfully blocked the paracrine stimulation of *Mx1* or *ISG15* expression downstream. We saw an increase of avH1N1/06 virus replication in the JAK/STAT inhibited group and a slightly reduced virus replication in the autocrine stimulated group. Therefore, we hypothesize that the avH1N1/06 is a weak stimulator of the innate immune response in swine cells, which allows the virus to replicate for a prolonged period of time, which opens the possibility for the further adaptation and virus reassortment. As to swH1N1/06, after ruxolitinib treatment, the similar kinetics of viral replication may indicate that the virus is either a strong modulator of the innate immune response or the NP protein itself may escape from the Mx1 oligomeric ring binding [59].

Our findings show that some avian influenza virus strains (especially H1N1 subtype) can infect pig airway epithelial cells without adaption. The HA binding affinity to specific linkage types of sialic acid is not the major determinant for avian IAV to infect swine. During the virus infection process, stimulation of host innate immune response does not prevent virus replication in the swine cells over a prolonged period of time. When the conditions are fulfilled, the swine airway may favor pigs over humans in generating reassortant viruses with gene segments from avian and mammalian IAVs. More information about the co-infection of differentiated airway epithelial cells by various influenza A viruses is required to get a better overview of the possible reassortant viruses that emerged in the human population and spread also in pigs.
